# Evaluation of the Sensitivity of Selected *Candida* Strains to Ozonated Water—An In Vitro Study

**DOI:** 10.3390/medicina58121731

**Published:** 2022-11-26

**Authors:** Anna Kuśka-Kiełbratowska, Rafał Wiench, Anna Mertas, Elżbieta Bobela, Maksymilian Kiełbratowski, Monika Lukomska-Szymanska, Marta Tanasiewicz, Dariusz Skaba

**Affiliations:** 1 Department of Periodontal Diseases and Oral Mucosa Diseases, Faculty of Medical Sciences in Zabrze, Medical University of Silesia, 40-055 Katowice, Poland; 2 Chair and Department of Microbiology and Immunology, Faculty of Medical Sciences in Zabrze, Medical University of Silesia, 40-055 Katowice, Poland; 3 Conservative Dentistry and Endodontics Clinic, General Dentistry Clinic, Academic Centre of Dentistry, 41-902 Bytom, Poland; 4 Department of General Dentistry, Medical University of Lodz, 251 Pomorska St., 92-213 Lodz, Poland; 5 Chair and Department of Conservative Dentistry with Endodontics, Faculty of Medical Sciences in Zabrze, Medical University of Silesia, 40-055 Katowice, Poland

**Keywords:** oral surgery, oral candidiasis, ozonated water, MTT, DMSO

## Abstract

(1) *Background and Objectives:* Oral candidiasis has increased significantly in recent years. Increasingly, we encounter treatment difficulties related to drug resistance. Therefore, it is necessary to search for other therapies such as ozone therapy, which has antimicrobial activity. The aim of this study was to determine the sensitivity of selected *Candida* strains to ozonated water based on concentration and contact time (2) *Methods:* The sensitivity of *Candida* strains to ozonated water with a concentration of 5 µg/mL, 30 µg/mL, and 50 µg/mL was assessed using Mosmann’s Tetrazolium Toxicity (MTT) assay. Statistical differences were assessed by the analysis of variance (ANOVA) and the Newman-Keuls post-hoc test. A *p-*value of ≤0.05 was considered to indicate a statistically significant difference. (3) *Results:* In all the strains and research trials, the number of viable cells was reduced by ozonated water. The reduction depended on the exposure time and concentration of ozonated water. The highest percentage reduction (34.98%) for the tested samples was obtained for the *C. albicans* strain after 120 s of exposure at the highest concentration-50 µg/mL. (4) *Conclusions:* The selected strains of *Candida* spp. were sensitive to ozonated water at all tested concentrations (5 µg/mL, 30 µg/mL, and 50 µg/mL). The sensitivity of strains to ozonated water increased with concentration and application time. Moreover, the sensitivity of *Candida* strains to ozonated water is comparable to that of 0.2% chlorhexidine gluconate.

## 1. Introduction

*Candida* fungi are part of the physiological saprophytic flora of the human oral cavity and are found in up to 25–65% of patients [1,2]. However, under certain conditions, namely disturbances of the balance and homeostasis of the organism or the incompetence of the host immune system, opportunistic infections and the development of candidiasis may occur. Due to the numerous and mutually overlapping risk factors for infection occurrence, the number of infections with *Candida* fungi has dramatically increased recently [3]. In clinical practice, doctors encounter increasingly huge difficulties in choosing the right pharmacological treatment because of reduced drug sensitivity or even complete resistance to fungi. Such a situation urged the search for new methods and substances with antifungal properties in order to supplement or even replace conventional therapy [4,5]. Alternative methods include essential oils (oregano, lemongrass, tea tree) [6,7,8,9,10], nano-metal colloidal solutions (silver, copper, gold) [11,12,13], antimicrobial photodynamic therapy (aPDT) (a type of light therapy that causes irreversible damage to target cells) [14,15,16], and ozone therapy (in various gaseous forms, water solutions, or ozonated oils) [17,18,19,20].

There are two forms of oxygen naturally occurring in the Earth’s atmosphere: molecular oxygen (O_2_), consisting of two oxygen atoms, and the allotropic form found in the stratosphere, ozone (O_3_), consisting of three oxygen atoms. O_3_ is a pale blue gas with a pungent, characteristic smell. It is heavier than air; therefore, as it moves from the higher layers of the atmosphere, it naturally cleans the air of pollutants. The solubility of ozone in water is 10-fold greater than that of diatomic oxygen, which decreases with increasing water temperature. The half-life of ozone in distilled water at room temperature is assumed to be approximately 30 min. It decomposes into a diatomic oxygen (O_2_) and an active singlet oxygen (^1^O_2_) molecule, whose strong oxidizing properties (resulting from a high redox potential) make ozone one of the strongest oxidants among substances with a disinfecting effect; its redox potential is respectively 2.07 V for ozone, 1.76 V for hydrogen peroxide, and 1.45 V for chloric acid (I) [21,22]. As a result, ozone has a wide therapeutic window, and therefore gram-positive bacteria, gram-negative bacteria, viruses, fungi, and vegetative cells are susceptible to its effects [23,24]. Its antimicrobial action is based on damaging the cell wall and subsequent destruction of the lipids in the cell membrane, causing an increase in its permeability and breakdown. Oxidized radicals act on fungi of the genus *Candida* by destabilizing the cell membrane and damaging cytoplasmic organelles. They also interact with purine and pyrimidine bases, damaging the genetic material, disrupting enzyme systems, and leading to the destruction of the cell [24].

It is worth emphasizing that ozone has many applications in dentistry. For example, ozone can be used to disinfect carious cavities and the root canal system before the final filling, and due tits deep penetration into the dentinal tubules, ozone enables more thorough decontamination. Additionally, ozone can be applied to treat dentine hypersensitivity by opening dentinal tubules and enhancing the penetration of calcium or fluorine ions, ensuring their long-term retention [23,25]. Regular application of low doses of ozone, alone or in combination with conventional therapy, improves the treatment effectiveness of mucosal diseases (aphthous erosions, cold sores, oral mycosis, lichen planus, and angular cheilitis). Tissue microscopic studies have shown that ozone therapy reduces inflammation and swelling and is useful in wound healing in soft tissues [26,27,28]. Additionally, a similar mechanism of action can be expected in the course of treatment of oral candidiasis [29].

A common form of antiseptic or medication used in the oral cavity are rinses. Mouth rinsing is most often recommended in the time range from 30 s to 2 min.

This study took these rinse requirements into account.

Therefore, the aim of the present study was to assess the sensitivity of selected *Candida* strains to ozonated water based on concentration and contact time.

The null hypothesis to be tested is that the contact time and concentration of ozonated water do not influence the sensitivity of selected *Candida* strains.

## 2. Materials and Methods

### 2.1. Organisms and Growth Conditions

This research was carried out on reference strains of *Candida* fungi from the American Type Culture Collection (ATCC, Manassas, VA, USA): *Candida albicans* ATCC 10,231, *Candida glabrata* ATCC 2001, and *Candida krusei* ATCC 34,135. These represent the most common strains causing oral candidiasis [30]. These strains were selected due to their high frequency of occurrence in patients treated for oral mycosis. However, they often show great difficulties in treatment due to reduced sensitivity to antifungal drugs. The approval of the bioethics committee was not required due to the use of reference strains from the ATCC collection.

Cultures of each strain were placed separately onto Sabouraud dextrose agar plates from bioMerieux (Marcy-l’Étoile, France), which contain peptone (10.0 g/L) and agar (8.0 g/L), and incubated in atmospheric air at 37 °C. After 24 h of incubation, a sample of colonies was removed from the surface of the plate and suspended in sterile physiological solution (0.9% NaCl). The number of viable cells in used suspension was counted in a Densi-La-Meter II densitometer (ERBA LACHEMA, Prague, Czech Republic) using the optical density of McFarland = 5.0.

### 2.2. Ozone Water

Ozonated water in a concentration of 5 µg/mL, 30 µg/mL, and 50 µg/mL was used, obtained with the use of an ATO-3 ozone therapy device (Metrum CryoFlex, Blizne Łaszczyńskiego, Warsaw, Poland). Distilled water (400 mL) was ozonated for 7 min. according to the manufacturer’s instructions. In order to preserve the properties of ozonated water, a new portion was prepared before each stage (due to the short half-life).

### 2.3. Chlorhexidine Digluconate

The 0.2% working solution was prepared immediately before the test by diluting a 2% aqueous solution of chlorhexidine digluconate (Cerkamed, Stalowa Wola, Poland) in water of pharmacopoeial purity. The choice of this substance is supported by studies showing its effectiveness against *Candida* spp. [26,31,32].

### 2.4. MTT Assay Protocol

The fungal cell viability was assessed using the MTT test. This is a quantitative study to determine the end product, formazan, which is formed by adding [3-(4,5-dimethyl-2-yl)-2,5-diphenyltetrazole] bromide (MTT reagent) from Sigma Chemical Company (St. Louis, MO, USA) to a fungus suspension previously treated with ozonated water. The MTT test assesses the activity of mitochondrial dehydrogenase, which is active only in living fungus cells. The amount of insoluble formazan formed is directly proportional to the number of viable cells. In order to dissolve the formazan crystals accumulating in the cells, it was necessary to use a solution of dimethylsulfoxide (DMSO) (Merck, Darmstadt, Germany). The concentration of the released dye was quantified in a universal microplate spectrophotometer at a wavelength of λ = 550 nm, comparing the values in the test samples to the control samples [33].

### 2.5. Experimental Groups and Inactivation of Candida spp. In Vitro

A total of 192 tests were prepared, 64 for each tested *Candida* strain. They were divided into the following groups: O_3_, the experimental groups in which the working yeast suspensions were treated with ozonated water (5 µg/mL, 30 µg/mL, and 50 µg/mL), and two control groups. In the positive control, the yeast working suspensions were treated with 0.2% chlorhexidine digluconate (CHX), while in the negative one (K) the working suspensions were exposed neither to ozonated water nor chlorhexidine solution. The experiments were performed under uniform experimental conditions in 8 independent replications (n = 8). In each of the experimental and control groups, the effects of different ozonated water concentrations and their exposure times on the reduction of viable cells were assessed as follows: 200 µL of yeast working suspensions were added to the wells of a 96-well plate. The plates were then centrifuged (at 2000 rpm per 5 min, in Jouan, France) to separate the microbial pellet from the supernatant, which was then drained. A volume of 200 µL of ozonated water was added to the test wells with a sterile pipette for a duration of 30, 60, and 120 s, respectively. A positive control (CHX) was carried out according to the above-described scheme, replacing ozonated water with freshly prepared 0.2% chlorhexidine digluconate. In the case of the control group (K), ozonated water was replaced with liquid Sabouraud medium (Marcy-l’Étoile, France). After the specified time, ozonated water, chlorhexidine, and Sabouraud’s liquid medium were removed, and 180 µL of bioMerieux Sabouraud liquid medium and 20 µL of MTT reagent were added to the wells. The plates with cells prepared in this way were incubated under aerobic conditions for 4 days at 37 °C. After this time, the samples were centrifuged (at 2000 rpm per 5 min, in Jouan, France), the supernatant was drained, and 200 µL of DMSO was added to the test cultures to extract insoluble formazan from the fungal cells. Then, 150 µL of the solution were collected and the absorbance was determined using an EonTM universal microplate spectrophotometer (BioTek Instruments, Winooski, USA) at a wavelength of λ = 550 nm. The color intensity of the test solution is proportional to the amount of formazan formed. Values of mitochondrial dehydrogenase activity determining the percentage of viable cells for the tested suspensions were calculated based on the following formula: cell viability = [AB/AK] × 100% (AB-absorbance of the experimental sample, AK-absorbance of the control sample).

### 2.6. Statistical Analysis

**Figure 1:**
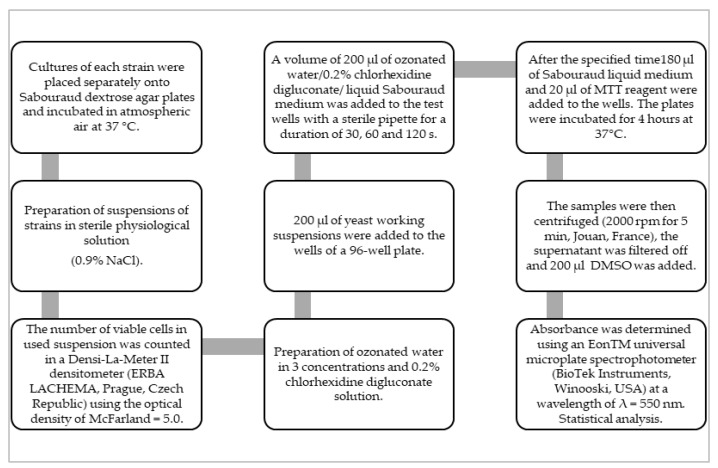
The study design.

The results were presented in histograms as the mean of the cell viability ± standard deviation. Statistical analysis was performed with the use of Excel 2013 by Microsoft and Statistica v. 7.1 PL by StatSoft Poland (Poland, Cracow). The convergence of the results with the normal distribution was assessed with the Shapiro-Wilk test. Statistical differences were assessed by the analysis of variance (ANOVA) and the Newman-Keuls post-hoc test. A *p*-value of ≤0.05 was considered to indicate a statistically significant difference (Figure 1).

## 3. Results

In the present study, the inhibitory effect of ozonated water at three concentrations (5 µg/mL, 30 µg/mL, and 50 µg/mL) on the viability of *Candida* spp. cells was assessed after 30, 60, and 120 s of incubation in experimental and control groups.

**Figure 2:**
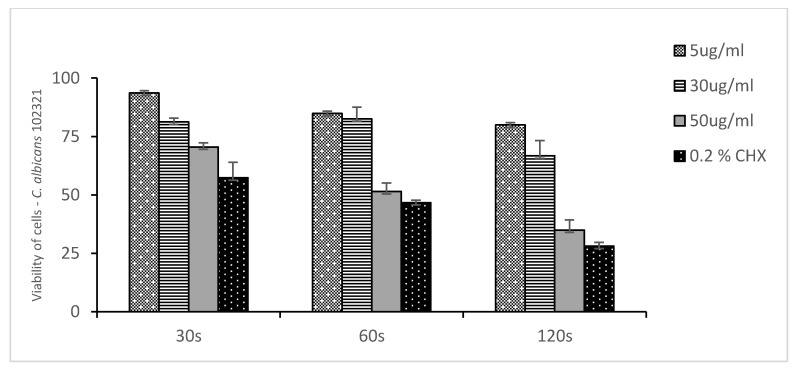
Viability of cells of *Candida albicans* ATCC 10,231 to ozonated water and chlorhexidine after three exposure times. n = 8 where n is the number of independent replications.

**Figure 3:**
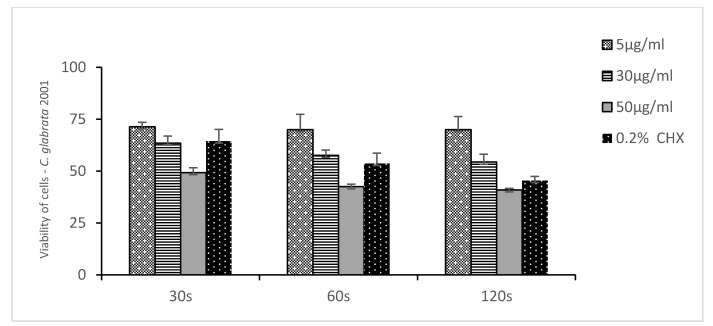
Viability of cells of *Candida glabrata* ATCC 2001 to ozonated water and chlorhexidine in three exposure times. n = 8.

**Figure 4:**
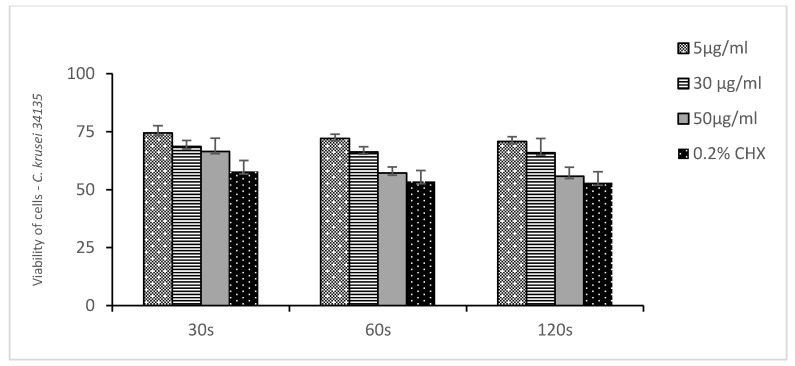
Viability of cells of *Candida krusei* ATCC 34,135 to ozonated water and chlorhexidine at three exposure times. n = 8.

**Figure 5:**
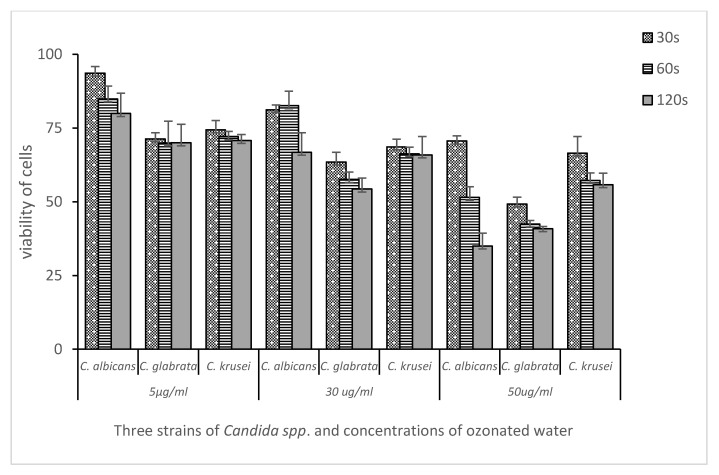
The effect of different exposure times (30 s–120 s) of ozonated water at three concentrations (5 µg/mL, 30 µg/mL, and 50 µg/mL) on cell viability (percentage of viability). Comparison between fungal strains *C. albicans* ATCC 10,231, *C. glabrata* ATCC 2001, and *C. krusei* ATCC 34,135.

**Figure 6:**
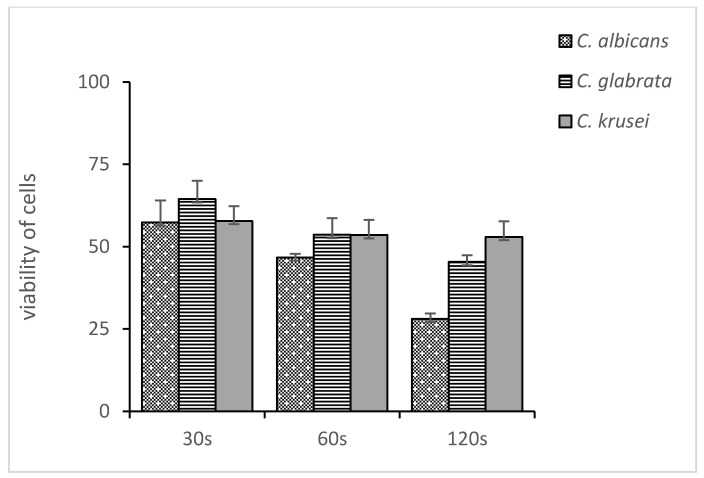
Effect of different exposure times on 0.2% chlorhexidine digluconate (30 s–120 s) on the number of viable cells (percentage of viability). Comparison between fungal strains *C. albicans ATCC* 10,231, *C. glabrata* ATCC 2001, and *C. krusei* ATCC 34,135.

Figure 2, Figure 3 and Figure 4 show the mean number of viable cells in percent and the standard deviation (M SD). In all strains, both in the test and the control groups (CHX), the number of live cells was reduced after exposure to ozonated water. The percentage reduction depended on the concentration of ozonated water and the time of exposure. The lowest viability for the test samples (50 µg/mL O_3_) was observed for the strain *C. albicans* ATCC 10,231 and amounted to 34.98% (Figure 2). Subsequently, for *C. glabrata* ATCC 2001, it was 40.85% (Figure 3), and for *C. krusei* ATCC 34,135 it was 55,79% (Figure 4). The lowest viability for all strains was found for the longest exposure time in ozonated water in the highest concentration. In the entire experiment, the lowest cell viability was obtained for *C. albicans* ATCC 10,231, as a result of the action of 0.2% chlorhexidine, and was 28.07% (Figure 2). Figure 5 shows the influence of different exposure times for ozonated water at three concentrations on the cell viability of the investigated strains. The study used a *p-*value where * *p* < 0.05, ** *p* < 0.01, and *** *p* < 0.001. The lowest concentration of ozonated water was the most effective against the *C. glabrata* ATCC 2001 strain, after 60 s of exposure; the viability of the strain amounted to 69.92%. However, statistically significant differences for this concentration and different exposure times have not been demonstrated. The greatest reduction in cell viability in the shortest time of ozonated water application was demonstrated for the *C. glabrata* ATCC 2001 strain; the cell viability of this strain amounted up to 49.25%. For this time, differences between individual concentrations of ozonated water for the described strain were statistically significant in each comparison (*p* < 0.001). On the other hand, for the longest exposure time to ozonated water- 50 µg/mL O_3_, the highest sensitivity was demonstrated for the strain *C. albicans* 10,231, where the cell viability was only 34.98%. The comparison between individual exposure times within this concentration of ozone water (50 µg/mL O_3_) showed a positive correlation between contact time with the active substance and the survival rate of *C. albicans* cells. Differences in this concentration of ozonated water for the described strain between individual exposure times were statistically significant in each comparison (*p* < 0.001) In 0.2% aqueous chlorhexidine digluconate the lowest cell viability at 30, 60 and 120 s was recorded for *C. albicans* ATCC 10,231 strain. It amounted to 57.36% for the shortest time and 28.07% for the longest application time (Figure 6). 

## 4. Discussion

Ozone therapy is a method that uses ozone in various forms and its properties. It has been successfully applied in medicine and dentistry for years [21].

The present results showed that the strain *C. albicans* ATCC 10,231 had the highest sensitivity during the longest exposure to ozonated water for 120 s. The cell viability of this strain was 34.98%, while the viability of the *C. krusei* ATCC 34,135 strain, in the same conditions, amounted to 55.79%. The viability of cells after a 120-s exposure to 0.2% chlorhexidine digluconate was found to be similar. It was 28.07% for *C. albicans* and 52.93% for the *C. krusei* strain. Additionally, the C. *albicans* strain is more sensitive to commonly used antiseptics, such as 0.2% chlorhexidine digluconate and ozonated water, than other tested strains. Accordingly, the null hypothesis tested in this study could be rejected. Similar results were obtained by Monzillo V. et al. [34], who analyzed the antifungal efficacy (*C. albicans*, *C. parapsilosis, C. glabrata, and C. tropicalis)* of GeliO_3_ ozonated oil compared to 0.2% chlorhexidine digluconate (Plakgel ^®^). Both products demonstrated antifungal activity against all *Candida* species tested. All species exhibited equal sensitivity to chlorhexidine. In the case of GeliO_3_, differences in sensitivity were found. The greatest ones were observed for *C. glabrata* and *C. albicans*, and the lowest for *C. parapsilosis* and *C. tropicalis* [34].

Other researchers confirmed the current study’s finding, demonstrating that the longer ozone application time, the more effective it is. De Faria et al. [35] assessed and compared the sensitivity to ozonated water of standard strains of *C. albicans* ATCC 18,804 and isolates collected from healthy students. The reduction in the number of strains progressed with increasing exposure time; however, complete reduction of colonies of the reference *C. albicans* strain occurred only after 5 min of exposure to ozone at a concentration of 3.3 mg/L. Moreover, freshly isolated strains of *C. albicans* showed higher resistance to ozone than the reference strains [35].

It is worth emphasizing that in the oral cavity there are pre-existing mixed bacterial and fungal biofilms. Moreover, fungi most often adhere to the surfaces occupied by bacteria. This phenomenon can be observed in the environments occupied by commensal flora, such as the oral cavity, and on the surfaces of dental materials, both in healthy and diseased individuals [36]. *Candida* spp. fungi can co-aggregate with other microorganisms living in the oral cavity, such as bacteria of the genera *Streptococcus*, *Staphylococcus*, *Actinomyces* and *Fusobacterium*, thus creating a species-diverse biofilm. Such interactions facilitate the survival and proliferation of fungi in the diverse microflora of the oral cavity. Currently, there are no studies available that would determine the sensitivity of microorganisms in mixed biofilms to ozonated water. However, based on the results for individual microorganisms, it can be presumed that it would also be effective against such a structure [20,22,25,36]. The use of an antifungal substance as medicine is often associated with many side effects and, in the case of the oral cavity, often with adverse effects on the surrounding tissues. In addition, the effects of water and ozonated oil were shown to be less toxic compared to other commonly used antiseptics [37]. The toxicity of ozone in various forms was evaluated for individual cells. Colombo M. et al. [37] proved the lack of cytotoxicity (MTT test) of ozonated olive oil and moderate to severe cytotoxicity of chlorhexidine digluconate (0.5% and 1%) against gingival fibroblasts (HGFs). Due to the fact that oral candidiasis often coexists with other mucosa pathologies, such as the severe form of lichen planus, it is desirable to have ozone stimulating the metabolic activity of fibroblasts, as well as immunomodulatory and anti-inflammatory effects based on the stimulation of the proliferation of immunocompetent cells and the synthesis of immunoglobulins. It also activates the phagocytic function of macrophages. In addition, it is believed that the effect of eliminating pain and inflammation is associated with the improvement in microcirculation and oxygenation of cells and tissues, as well as with the reduced production of pro-inflammatory cytokines and elimination of pain mediators [27,28].

The present results confirmed earlier reports that showed high activity of ozonated water against yeasts of the genus *Candida* [35]. It is well known that *non-albicans* strains are less sensitive to ozone [34]. The effectiveness of the therapy seems to be dependent on the concentration of the ozone solution used and the exposure time. Single laboratory tests also show low toxicity to epithelial cells and fibroblasts [37]. All this points to the potential possibility of using ozone (in the form of water or oil solutions) in local therapy not only on the oral mucosa but also on the mucosa in other parts of the body, and on the skin and its appendages. The ozone activity also shows activity against bacteria, thus the application of ozonated water solutions for mixed bacterial and fungal infections can be considered. However, there are no clinical studies on this subject in the available literature.

For this reason, the laboratory experience gained should be used to plan and conduct further studies and allow for the use of this substance as a supportive therapy or monotherapy.

The great advantage of this research, which contributes to its innovativeness and novelty, is the use of ozone water prepared ex tempore at particularly selected times of its impact. Exposure times were chosen to reflect the effectiveness of antiseptics with proven anti-fungal activity. Such studies using these conditions had not been conducted before.

One of the main goals of this study was to use the shortest possible time of exposure to ozonated water. In clinical terms, ozonated water would be used as a mouthwash. The time range of 30 to 120 s is most often recommended for mouthwash with other antibacterial substances. It is also a real and practical time that can be used by the patient in the dental office and at home. For this reason, our main goal was to test the sensitivity of the assessed strains to ozonated water under these conditions and exposure times.

In our study, we assessed the number of live cells after a single treatment. As with most antifungal substances, a single application is unlikely to be sufficient. In the future, it should be considered to conduct research defining a treatment regimen (number of days and frequency of use) using ozonated water, allowing for complete eradication. The presented study may be a preparation for further studies involving our patients. In the study, we plan to eliminate microorganisms not only from the mucous membrane but also from the denture plate. A limitation of the present study was the fact that only three concentrations were investigated; therefore, future research should include more concentrations of ozonated water. However, it should be acknowledged that it was a limitation of the device used for the study. Moreover, additional antiseptics as a control should be examined. The present study used only three reference strains of *Candida* fungi, thus more fungi strains were needed, including mixtures of several Candida strains and those collected from patients.

## 5. Conclusions

Within the limitations of this study, it can be concluded that selected strains of *Candida* spp. were sensitive to ozonated water at all tested concentrations (5 µg/mL, 30 µg/mL, and 50 µg/mL). The sensitivity of strains to ozonated water increased with concentration and application time. Moreover, the sensitivity of *Candida* strains to ozonated water is comparable to that of 0.2% chlorhexidine gluconate.

## Data Availability

Not applicable.

## References

[1] Giacobbe D.R., Maraolo A.E., Simeon V., Magnè F., Pace M.C., Gentile I., Chiodini P., Viscoli C., Sanguinetti M., Mikulska M. (2020). Changes in the relative prevalence of candidaemia due to non-albicans Candida species in adult in-patients: A systematic review, meta-analysis and meta-regression. Mycoses.

[2] Nobile C.J., Johnson A.D. (2016). *Candida albicans* Biofilms and Human Disease. Annu. Rec. Microbiol..

[3] Vila T., Sultan A.S., Montelongo-jauregui D. (2020). Oral Candidiasis: A Disease of Opportunity. J. Fungi.

[4] Rodrigues C.F., Rodrigues M.E., Henriques M.C.R. (2018). Promising Alternative Therapeutics for Oral Candidiasis. Curr. Med. Chem..

[5] Welk A., Zahedani M., Beyer C., Kramer A., Müller G. (2016). Antibacterial and antiplaque efficacy of a commercially available octenidine-containing mouthrinse. Clin. Oral Investig..

[6] Soares I.H., Loreto S., Rossato L., Mario D.N., Venturini T.P., Baldissera F., Santurio J.M., Alves S.H. (2015). In vitro activity of essential oils extracted from condiments against fluconazole-resistant and -sensitive Candida glabrata. J. Mycol. Med..

[7] Srivatstava A., Ginjupalli K., Perampalli N.U., Bhat N., Ballal M. (2013). Evaluation of the properties of a tissue conditioner containing origanum oil as an antifungal additive. J. Prosthet. Dent..

[8] Ninomiya K., Hayama K., Ishijima S.A., Maruyama N., Irie H., Kurihara J., Abe S. (2013). Suppression of inflammatory reactions by terpinen-4-ol, a main constituent of tea tree oil, in a murine model of oral candidiasis and its suppressive activity to cytokine production of macrophages In Vitro. Biol Pharm Bull..

[9] Szweda P., Gucwa K., Kurzyk E., Romanowska E., Dzierżanowska-Fangrat K., Zielińska Jurek A., Kuś P.M., Milewski S. (2015). Essential Oils, Silver Nanoparticles and Propolis as Alternative Agents Against Fluconazole Resistant Candida albicans, Candida glabrata and Candida krusei Clinical Isolates. Indian J. Microbiol..

[10] Dalwai S., Rodrigues S.J., Baliga S., Shenoy V.K., Shetty T.B., Pai U.Y., Saldanha S. (2016). Comparative evaluation of antifungal action of tea tree oil, chlorhexidine gluconate and fluconazole on heat polymerized acrylic denture base resin—An in vitro study. Gerodontology.

[11] Jebali A., Hajjar F.H.E., Pourdanesh F., Hekmatimoghaddam S., Kazemi B., Masoudi A., Daliri K., Sedighi N. (2014). Silver and gold nanostructures: Antifungal property of different shapes of these nanostructures on Candida species. Med. Mycol..

[12] Ingle A.P., Duran N., Rai M. (2014). Bioactivity, mechanism of action, and cytotoxicity of copper-based nanoparticles: A review. Appl. Microbiol. Biotechnol..

[13] Nam K.Y. (2011). In vitro antimicrobial effect of the tissue conditioner containing silver nanoparticles. J. Adv. Prosthodont..

[14] Wiench R., Nowicka J., Pajaczkowska M., Kuropka P., Skaba D., Kruczek-Kazibudzka A., Kuśka-Kiełbratowska A., Grzech-Leśniak K. (2021). Influence of incubation time on ortho-toluidine blue mediated antimicrobial photodynamic therapy directed against selected Candida strains—An in vitro study. Int. J. Mol. Sci..

[15] Wiench R., Skaba D., Matys J., Grzech-Leśniak K. (2021). Efficacy of Toluidine Blue—Mediated Antimicrobial Photodynamic on *Candida* spp. A systematic Review. Antibiotics.

[16] Wiench R., Skaba D., Stefanik N., Kępa M., Gilowski Ł., Cieślar G., Kawczyk-Krupka A. (2019). Assessment of sensitivity of selected Candida strains on antimicrobial photodynamic therapy using diode laser 635 nm and toluidine blue—In vitro research. Photodiagnosis Photodyn. Ther..

[17] Kumar T., Arora N., Puri G., Aravinda K., Dixit A., Jatti D. (2016). Efficacy of ozonized olive oil in the management of oral lesions and conditions: A clinical trial. Contemp. Clin. Dent..

[18] Khatri I., Moge G., Kumar N.A. (2015). Evaluation of effect of topical ozone therapy on salivary Candidal carriage in oral candidiasis. Indian J. Dent. Res..

[19] Sechi L., Lezcano I., Nunez N., Espim M., Pinna A., Molicotti P., Dupre I., Microbiologia I. (2001). Antibacterial activity of ozonized sunfower oil (Oleozon). J. Appl. Microbiol..

[20] Lezcano I., Nuñez N., Espino M., Gómez M. (2000). Antibacterial activity of ozonized sunflower oil, oleozon, against *Staphylococcus aureus* and *Staphylococcus epidermidis*. Ozone Sci. Eng..

[21] Garg R. (2009). Ozone: A new face of dentistry. Internet J. Dent. Sci..

[22] Tiwari S., Avinash A., Katiyar S., Iyer A.A. (2016). Dental applications of ozone therapy : A review of the literature. Saudi J. Dent. Res..

[23] Kumar P., Tyagi P., Bhagawati S., Kumar A. (2014). Current interpretations and scientific rationale of the ozone usage in dentistry: A systematic review of literature. Eur. J. Gen. Dent..

[24] Zeng J., Lu J. (2018). Mechanisms of action involved in ozone-therapy in skin diseases. Int. Immunopharmacol..

[25] Suh Y., Patel S., Kaitlyn R., Gandhi J., Joshi G., Smith N.L., Khan S.A. (2019). Clinical utility of ozone therapy in dental and oral medicine. Med. Gas Res..

[26] Gupta G., Mansi B. (2012). Ozone therapy in periodontics. J. Med. Life.

[27] Naik S.V., Kohli S., Zohabhasan S., Bhatia S. (2016). Ozone—A Biological Therapy in Dentistry-Reality or Myth. Open Dent. J..

[28] Colombo M., Gallo S., Garofoli A., Poggio C., Arciola C.R., Scribante A. (2021). Ozone gel in chronic periodontal disease: A randomized clinical trial on the anti-inflammatory effects of ozone application. Biology.

[29] Cardoso M.G., de Oliveira L.D., Koga-Ito C.Y., Jorge A.O.C. (2008). Effectiveness of ozonated water on Candida albicans, Enterococcus faecalis, and endotoxins in root canals. Oral Surg. Oral Med. Oral Pathol. Oral Radiol. Endodontol..

[30] Falagas M.E., Roussos N., Vardakas K.Z. (2010). Relative frequency of albicans and the various non-albicans *Candida* spp among candidemia isolates from inpatients in various parts of the world: A systematic review. Int. J. Infect. Dis..

[31] Kolliyavar B., Shettar L., Thakur S. (2016). Chlorhexidine: The Gold Standard Mouth Wash. J. Pharm. Biomed. Sci..

[32] Nogales C.G., Ferrari P.H., Olszewer K.E. (2008). Ozone Therapy in Medicine and Dentistry. J. Contemp. Dent. Pract..

[33] Kumar P., Nagarajan A., Uchil P. (2018). Analysis of Cell Viability by the MTT Assay. Cold Spring Harb. Protoc..

[34] Monzillo V., Lallitto F., Russo A., Poggio C., Scribante A., Arciola C.R., Bertuccio F.R., Colombo M. (2020). Ozonized gel against four Candida species: A pilot study and clinical perspectives. Materials.

[35] De Faria I.D.S., Ueno M., Yumi Koga-Ito C., Urrichi Irrazabal W., Balducci I., Jorge A.O.C. (2005). Effects of ozonated water on Candida albicans oral isolates. Braz. J. Oral Sci..

[36] Lohse M.B., Gulati M., Johnson A.D., Nobile C.J. (2017). Development and regulation of single- and multi-species *Candida albicans* biofilms. Nat. Publ. Gr..

[37] Colombo M., Ceci M., Felisa E., Poggio C., Pietrocola G. (2018). Cytotoxicity evaluation of a new ozonized olive oil. Eur. J. Dent..

